# Effects of Two Different Levels of Dietary Protein on Body Composition and Protein Nutritional Status of Growing Rats

**DOI:** 10.3390/nu4091328

**Published:** 2012-09-20

**Authors:** Julio Tirapegui, Sandra Maria Lima Ribeiro, Ivanir Santana de Oliveira Pires, Marcelo Macedo Rogero

**Affiliations:** 1 Department of Food Science and Experimental Nutrition, Faculty of Pharmaceutical Sciences, University of São Paulo, São Paulo, SP, CEP 05508-000, Brazil; Email: tirapegu@usp.br (J.T.); ivanirpires@yahoo.com.br (I.S.O.P.); 2 School of Arts, Sciences and Humanities, University of São Paulo, São Paulo, SP, CEP 03828-000, Brazil; Email: smlribeiro@usp.br; 3 Department of Nutrition, School of Public Health, University of São Paulo, São Paulo, SP, CEP 01246-904, Brazil

**Keywords:** dietary protein, protein nutritional status, growth, body composition

## Abstract

This study aimed to investigate the effect of a high-protein diet on growth, body composition, and protein nutritional status of young rats. Newly-weaned Wistar rats, weighing 45–50 g, were distributed in two experimental groups, according to their diets, which contained 12% (G12) or 26% protein (G26), over a period of 3 weeks. The animals were euthanized at the end of this period and the following analyses were performed: chemical composition of the carcass, proteoglycan synthesis, IGF-I concentration (serum, muscle and cartilage), total tissue RNA, protein concentration (muscle and cartilage) and protein synthesis (muscle and cartilage). The high-protein diet was found to result in a higher fat-free mass and lower fat mass in the carcass, with no difference in growth or protein nutritional status.

## 1. Introduction

Body growth, under different conditions, depends on coordinated regulation of hormonal factors, alongside nutritional supply [1,2]. Growth processes require nutrient use to be directed towards cell multiplication, energy storage and skeletal elongation [3,4], and proteins play a key role in these processes. Protein and nitrogen homeostasis, in addition to nitrogen balance, are achieved through a complex equilibrium between protein intake and amino acid metabolism, which includes whole body protein turnover, amino acid oxidation, urea production and nitrogen excretion during fasting and feeding periods of the day [5]. 

In order to achieve optimal effects of diet on growth and body composition, optimal proportions among macronutrients has been extensively discussed, without a definite consensus [6,7,8,9]. Studies, mainly performed with adults, have demonstrated advantageous consequences of high-protein diets, such as reduction of body fat [8,10], decrease in lipogenesis [11] and boosting of energy expenditure, protein balance and satiety [12]. On the other hand, it is not clear whether a high protein intake might further enhance the lean body mass [8,13]. 

Despite a number of published articles investigating the effects of different protein levels in adults, few studies have been performed during the growing stages. Previous studies of ours, employing growing animals, investigated the effects of different levels of dietary protein on some aspects of growth and protein nutritional status. First, we investigated the effect of two levels of protein in the diet (0.5% and 12%) and we concluded that 0.5% jeopardized growth and protein nutritional status, whilst 12% protein seemed to be enough to allow appropriate nutritional status [14]. Subsequently, we compared different levels of dietary protein (14%, 21% and 28%) in growing animals and we speculated that a protein level of above 21% and below 28% protein could provide benefits in some variables related to growth and protein nutritional status [15]. 

The above information suggests that despite the fact that a 12% protein level can result in an acceptable nutritional status, a relatively high level of intake could provide optimal conditions of body growth and body composition in growing animals. As such, the present study aimed to investigate the effects of a 26% protein diet, compared to a 12% protein diet, on growth, body composition and some biochemical markers of protein nutritional status in growing rats.

## 2. Experimental Section

### 2.1. Animals and Experimental Diets

Newly-weaned Wistar rats (*n* = 16), 21-days old, weighing 40–50 g, were obtained from the Animal Laboratory of the Faculty of Pharmaceutical Sciences at the University of São Paulo. The local Ethics Committee approved all experimental procedures, and the guidelines for animal care and use were followed according to the Declaration of Helsinki.

The animals were individually housed in stainless-steel cages in the animal house at a constant temperature (22 °C ± 2 °C) and humidity (55% ± 10%), on a 12-h light/12-h dark cycle (lights on at 07:00 a.m.). All animals were weighed daily at 08:00 a.m. At that time, the water and the non-ingested food were weighed and withdrawn from the cages; the new food was weighed and offered to the animals in a specific receptacle inside the cages. During the remaining time, the animals received food and water *ad libitum*. As such, this schedule ensured that animals of all groups had the same time of feeding.

### 2.2. Experimental Procedure

The study was conducted on two experimental groups of eight rats each respectively, feeding *ad libitum* diets containing 12% (G12) and 26% protein (G26) over a period of 3 weeks. The diets were elaborated based on previous studies from our group [16,17,18] (Table 1).

**Table 1:** Composition of experimental diets ^1^.

Ingredients (g/kg)	G12	G26
Casein ^2^	141	305.5
Sucrose	100	100
Fiber	50	50
Corn oil	50	50
Mineral mixture	35	35
Vitamin mixture	10	10
dl-Methionine	1.8	0.7
Choline bitartrate	2	2
Cornstarch	610.2	446.8
Total weight	1000	1000
Macronutrient composition ^3^		
Carbohydrate	73.56	56.63
Protein	14.79	31.71
Lipid	11.65	11.65

^1 ^Isocaloric diets providing 1613 kJ/100 g (386 kcal/100 g); ^2 ^Casein contains 85% protein; ^3 ^Percentage of the total metabolizable energy.

On the day after the last day of the experiment (week 3), following 8h of fasting, the animals were euthanized by decapitation (between 08:00 a.m. and 11:00 a.m.). Before euthanasia, the animals were injected intraperitoneally with 0.4 µCi ^35^S-sodium sulfate (Sigma, St. Louis, MO, USA) (60 min before) and 0.2 µCi ^3^H-phenylalanine (Sigma, St. Louis, MO, USA) (15 min before) per gram body weight for determination of proteoglycan and protein synthesis, respectively [16,17].

Blood, collected by inversion of the animal, was centrifuged and plasma was stored at −80 °C for later biochemical determination. The gastrocnemius and soleus muscles were weighed, submerged in liquid nitrogen and stored at −80 °C for later determinations. The tibia of both legs were dissected, and the hyaline cartilage was then extracted manually using a surgical knife, cleaned of all visible traces of muscle and tendons and cut into small pieces. The tibias’ lengths were measured with a Norma caliper and reported as millimeter.

### 2.3. Analytical Method and Procedures

*Total RNA and Protein Concentration:* Protein content was measured in muscle and cartilage by the Folin method of Lowry *et al.* [19], using bovine albumin (Sigma, St. Louis, MO, USA) as standard. RNA was measured in muscle and cartilage homogenate by the method of Munro and Fleck [20,21]. 

*Protein Synthesis:* Cartilage and muscle protein synthesis (Ks, % day) was measured “*in vivo*” using a large-dose phenylalanine method [16]. The large dose of amino acids results in a rapid rise in the specific radioactivity of free phenylalanine in tissues to values close to the value in plasma, followed by a slow but linear fall. This enables the rate of protein synthesis to be calculated from measurements of the specific radioactivity of free and protein-bound phenylalanine in tissues during a 10 min period after injection of radioisotope. Labeled compounds (Amersham, Bucks, UK) were evaporated to dryness before being dissolved in the appropriate injection solution. L-Tyrosine decarboxylase, B-phenetylamine and leucylalanine were purchased from Sigma Chemical Co., Poolet, Dorset, UK, and ninhydrin was purchased from BDH, Poole, Dorset, UK.

*Proteoglycan Synthesis:* A sample of gastrocnemius muscle was weighed, pulverized and homogenized in ice-cold 10% trichloroacetic acid (TCA) (Merck Chemicals, Massachusetts, USA), washed twice with 10% TCA and with perchloric acid (Merck Chemicals, Massachusetts, USA) to remove any residual free ^35^SO_4_, and solubilized in 2 mL 90% (w/v) formic acid (Merck Chemicals, Massachusetts, USA), after incubation at 90 °C overnight. ^35^S radioactivity was measured in a Beckman LS-150 liquid scintillation counter, and expressed as dpm/mg tissue. The hyaline cartilage of both legs were weighed accurately into a polypropylene test tube, homogenized twice in ice-cold acid-ethanol (1.25 mL/50 mg) to give a final yield of 2.5 mL supernatant/50 mg tissue for the measurement of tissue IGF-I, and the precipitate was then treated with 10% TCA as for muscle [17].

*IGF-1 Extraction:* Frozen muscle was pulverized between aluminum plates that had been previously immersed in liquid nitrogen. The pulverized samples were placed in polypropylene tubes, also previously immersed in liquid nitrogen, and an 85% ethanol solution and a 15% 6 M HCl solution were added (Merck Chemicals, Massachusetts, USA). IGF-I was extracted twice from 100 mg pulverized tissue in 2.5 mL ice-cold acid ethanol (87.5% (v/v) ethyl-alcohol and 12.5% (v/v) HCl, (2 mol/L)) and the combined supernatants were evaporated to dryness under vacuum, dissolved in 600 µL NaOH (0.5 mol/L) neutralized with HCl (0.5 mol/L) centrifuged and the supernatant dried again under vacuum. The extract was reconstituted in 300 µL assay buffer and then assayed for IGF-I. A similar procedure was used for the acid-ethanol supernatant of the tibia epiphysis, prepared as described above [22]. These extracts and plasma (also submitted to acid-ethanol extraction to dissociate and separate IGF-I from binding proteins) were analyzed from the IGF-I by radioimmunoassay (RIA). The neutralized supernatant was incubated with a rabbit polyclonal anti-human IGF-I first antibody and labeled with human IGF-I (^125^I-labelled IGF-I) (Sigma, Norbiton, Surrey, UK). After an overnight incubation, the IGF-I-antibody complex was precipitated with a polyethylene glycol (PEG)-globulin mix, which acted as a non-specific second antibody (125 mL 40% (w/v) PEG-6000/50 mL 0.2 mol Tris-Cl/L buffer, pH 8.5/25 mL distilled water/0.3 rabbit gamma-globulin (Sigma, Norbiton, Surrey, UK)). 

*Radioimmunoassays (RIA):* Serum Insulin and IGF-I, as well as muscle and cartilage IGF-I were determined by radioimmunoassay (RIA), according to Herbert *et al.* [23] and Daughaday *et al.* [22], respectively. 

*Serum Albumin and Protein Concentrations: *Protein and serum albumin were assayed by a colorimetric method using commercial kits (CELM, São Paulo, Brazil).

*Chemical Composition of the Carcass: *The chemical composition of the carcasses has been reported in our previous study [24]. Briefly, the whole carcass was dried in a ventilated oven to determine the moisture composition. Subsequently, we analyzed lipid (by solvent extraction), protein and ash contents.

### 2.4. Statistical Methods

All the results are presented as means ± standard deviations. After the establishment of data normality (by Levene’s test), the comparison between G12 and G26 was made using the Student *t*-test for independent samples, with the level of significance set at 5%.

## 3. Results

Table 2 depicts food and protein intake, and biometric variables of experimental animals. There were no significant differences between groups with regard to body weight or bone length. In addition, the daily and total food intake did not differ between the groups.

**Table 2:** Food and protein intake and biometric variables of experimental groups^1,2^.

Variables	G12	G26
Total food intake (g)	356 ± 29	331 ± 18
Food intake (g/day)	16.2 ± 1.3	15.1 ± 0.8
Protein intake (g/day)	1.9 ± 0.2	3.9 ± 0.2 *
Initial body weight (g)	48 ± 2	49 ± 2
Final body weight (g)	182 ± 20	185 ± 11
Body weight gain (g)/g food	0.38 ± 0.02	0.41 ± 0.03
Body weight gain(g)/g protein intake	3.36 ± 0.18	1.6 ± 0.02 *
Tibia length (mm)	33.2 ± 0.7	33.8 ± 0.6

^1^ Eight rats per group; ^2^ Mean ± SD; * Significantly different from G12 (*P* < 0.05).

Table 3 shows plasma biochemical variables. None of the serum markers that were measured to indicate protein nutritional status presented any significant difference between the groups. Similarly, the muscle weight, biochemical analysis (Table 4) and cartilage analysis (Table 5) did not demonstrate any significant differences between the groups.

**Table 3:** Biochemical variables for plasma of experimental groups ^1,2^.

Parameter	G12	G26
Insulin (μU/mL)	59.7 ±16.1	59.9 ± 11.6
IGF-I (ng/mL)	573 ± 67	627 ± 89
Albumin (g/dL)	3.5 ± 0.3	3.7 ± 0.1
Total protein (g/dL)	5.6 ± 0.3	5.4 ± 0.2

^1^ Eight rats per group; ^2^ Mean ± SD.

**Table 4:** Growth and protein synthesis in gastrocnemius muscle tissue ^1,2^.

Parameter	G12	G26
Muscle weight (mg)	843 ± 112	832 ± 38
IGF-I (ng/g)	11.0 ± 1.0	12.5 ± 0.9
Proteoglycan synthesis (dpm/mg)	1073 ± 62	968 ± 81
Protein (mg/100 mg)	12.9 ± 1.4	12.9 ± 1.1
RNA (μg/100 mg)	132.4 ± 9.1	123.4 ± 6.1
Protein synthesis (% day)	12.8 ± 0.8	11.9 ± 1.1

^1^ Eight rats per group; ^2^ Means ± SD.

**Table 5:** Growth and protein synthesis in cartilage tissue ^1,2^.

Parameter	G12	G26
Cartilage weight (mg)	91 ± 6	85 ± 9
IGF-I (ng/g)	39.2 ± 1.7	40.6 ± 4.3
Proteoglycan synthesis (dpm/g)	265 ± 66	270 ± 47
Protein (mg/100 mg)	8.6 ± 0.9	8.8 ± 0.6
RNA (μg/100 mg)	252 ± 17	248 ± 15
Protein synthesis (% day)	80.2 ± 8.1	76.9 ± 8.2

^1^ Eight rats per group; ^2^ Means ± SD.

Table 6 presents the results for the chemical composition of the animals’ carcasses. The higher protein intake (G26) resulted in higher lean mass, water and protein content and lower fat, both in absolute and percentage values. 

**Table 6:** Carcass composition of the experimental groups ^1,2^.

	Absolute values (g)		Percentage of total carcass weight (%)	
	G12	G26	G12	G26
Lean mass	164.3 ± 2.6	170.5 ± 2.1 *	90.3 ± 1.4	92.2 ± 1.1 *
Moisture	113.6 ± 2.7	118.9 ± 1.7 *	62.4 ± 1.5	64.3 ± 0.9 *
Fat	17.7 ± 2.6	14.5 ± 2.1 *	9.7 ± 1.4	7.8 ± 1.1 *
Protein	33.5 ± 1.5	35.6 ± 6.3 *	18.4 ± 0.8	19.3 ± 0.3 *

^1^ Eight rats per group; ^2^ Mean ± SD; * Significantly different from G12 (*P* < 0.05).

## 4. Discussion

The present study aimed to investigate whether two different levels of protein in the diet result in differences in growth, body composition and protein nutritional status in growing rats. We found that a 26%-protein diet resulted in a higher protein content and lower fat content in the animal carcasses, but did not alter any biochemical variables related to protein nutritional status.

The finding of reduced fat in the G26 carcasses is in keeping with some previously published studies. Jean *et al.* [8] studied the effects of long-term adaptation to a high-protein diet (50 g/100 g dry matter) compared to a normal diet (14 g/100 g dry matter) on body composition in rats. The authors observed that the reduction in body weight resulting from high protein diet was mainly due to a reduction in body fat mass, without any significant effects on lean body mass, signifying an increased lean/fat mass ratio. Additionally, Du *et al.* [25] determined the dose-response effect of different dietary protein percentages (2%, 5%, 8%, 10%, 15% *vs.* 20% casein) on food intake, body composition and energy balance. With the exception of 2% protein, the authors observed that the higher the level of dietary protein, the lower the proportion of fat incorporated into the carcass. 

With regard to the observation of a higher protein content and lower fat content in the G26 carcasses, it may be hypothesized that the specific orientation of energy metabolism, with amino acids as the principal energy substrate, may lead to these alterations. Indeed, the diversion of amino acids to catabolic pathways and gluconeogenesis is generally associated with a higher thermogenic effect of these types of diet. It is known that protein has a relatively higher thermogenic effect (20%–30%), when compared to carbohydrate (5%–10%) or fat (0–3%). This can be partly explained by the fact that the body has no flexible storage capacity for an excess intake of amino acids, which are therefore actively oxidized or eliminated. Indeed, although protein oxidation and urea synthesis require energy, it is well known that one of the most costly protein-metabolic processes is protein synthesis [21,22]. The precise mechanisms involved in such diversion, however, into the various metabolic pathways, require further elucidation [8].

The ingestion of a high-protein meal produces acute effects, known to facilitate body fat regulation, such as the stimulation of protein synthesis and energy expenditure. Protein intake stimulates protein synthesis and this effect could blunt its potential effect on fat loss [26,27]. 

It is important to note that, to achieve 26% of protein in the diet, we reduced the amount of carbohydrates. *De novo lipogenesis*, a highly active process in rodents, could be stimulated by an excess flow of glucose to glycolysis and could, consequently, enhance the hepatic acetyl-CoA pool [28]. These increases in substrate availability could culminate in an enhancement of total body fat, since they are responsible for lipogenesis. Thus, reducing carbohydrates in the diet (as occurred for G26) could reduce, or slow down, these lipogenic reactions, improving lean body mass and reducing body fat.

Serum markers of protein nutritional status (serum albumin, IGF-I, insulin and total protein), as well as muscle and bone markers of growth (proteoglycan synthesis, protein and RNA content, and protein synthesis), were not modified by the higher protein intake. When comparing with other studies, it may be noted that all these biomarkers are modified much more in response to a low protein intake than to a high protein intake. In this context, Yahya *et al. *[29] investigated the effect of protein deficiency by *ad libitum* feeding of different levels of protein in the diet (20%, 7%, 3.5% and 0.5%); authors showed that the administration of a diet that provided 0.5% protein to rats was able to reduce the serum albumin level, when compared with the levels of 7% or 20% of protein in the diet. Gomes *et al.* [30] showed that a protein-free diet resulted in an expressive reduction in serum and muscle IGF-I concentration. Yahya *et al.* [17] reported inhibition of protein synthesis, RNA activity and RNA content in the muscle and cartilage of the tibia epiphysis of rats submitted to low-protein diets. According to these investigators, bone IGF-I specifically regulates the mechanisms of protein and proteoglycan synthesis in cartilage. In muscle, however, protein synthesis may be mainly regulated by the action of insulin.

It is important, at this point, to highlight some limitations of the present study. We investigated the effects of a high-protein diet using an animal model, which is certainly limited in its application to humans, particularly in children and adolescents. In addition, although we did not identify any deleterious modification in protein nutritional status as result of the slightly high protein diet, it is necessary to investigate these effects over long–term periods. Recent studies have investigated the modification of diet during childhood and the repercussions of these changes in adulthood health [31,32].

## 5. Conclusions

From our experimental conditions, it was possible to conclude that a diet composed of 26% protein improved a number of variables related to body composition, when compared with a 12% protein diet. However, this diet did not demonstrate significant advantages with regard to growth and protein nutritional status.
